# District Health Teams’ Readiness to Institutionalize Integrated Community Case Management in the Uganda Local Health Systems: A Repeated Qualitative Study

**DOI:** 10.9745/GHSP-D-19-00318

**Published:** 2020-06-30

**Authors:** Agnes Nanyonjo, Edmound Kertho, James Tibenderana, Karin Källander

**Affiliations:** a Malaria Consortium Uganda, Kampala, Uganda.; b Malaria Consortium, London, UK.; c Department of Public Health Sciences, Karolinska Institutet, Stockholm, Sweden.

## Abstract

**Introduction:**

Several countries have adopted integrated community case management (iCCM) as a strategy for improved health service delivery in areas with poor health facility coverage. Early implementation of iCCM is often run by nongovernmental organizations financed by donors through projects. Such projects risk failure to transition into programs run by the local health system upon project closure. Engagement of subnational health authorities such as district health teams (DHTs) is essential for a smooth transition.

**Methods:**

We used a repeated qualitative study design to assess the readiness of and progress made by DHTs in institutionalizing iCCM into the functions of locally decentralized health systems in 9 western Uganda districts. Readiness data were derived from structured group interviews with DHTs before iCCM policy adoption in 2010 and again in 2015. Progressive institutionalization achievements were assessed through key informant interviews with targeted DHT members and local government district planners in the same areas.

**Findings:**

In the readiness study, DHTs expressed commitment to institutionalize iCCM into the local health system through the development of district-specific iCCM activity work plans and budgets. The DHTs further suggested that they would implement district-led training, motivation, and supervision of community health workers; procurement of iCCM medicines and supplies; and advocacy activities for inclusion of iCCM indicators into the national health information systems. After iCCM policy adoption, follow-up study data findings showed that iCCM was largely not institutionalized into the local district health system functions. The poor institutionalization was attributed to lack of stewardship on how to transition from externally supported implementation to district-led programming, conflicting guidelines on community distribution of medicines, poor community-level accountability systems, and limited decision-making autonomy at the district level.

**Conclusion:**

Successful institutionalization of iCCM requires local ownership with increased coordination and cooperation among governmental and nongovernmental actors at both the national and district levels.

## Abstract (precis)

District health teams failed to transition from partner-supported integrated community case management (iCCM) programs to locally-run and fully-institutionalized programs. Successful iCCM institutionalization requires local ownership with increased coordination among governmental and nongovernmental actors at the national and district levels.

## Teaser

**Key Findings:**

- Despite district health teams reporting readiness and preparation to incorporate integrated community case management (iCCM) into their district health systems, they were unable to institutionalize most components into district-specific work plans.
- Barriers to institutionalization included the lack of stewardship from both Ministry of Health and implementing partners on how to transition from a partner-supported to a locally-run program, lack of agreement of national guidelines on community-level drug distribution, lack of integration of some iCCM indicators in the national health management information system, and limited central government funding.

**Key Implications:**

- District health teams and other local government administrative structures should work together to increase community acceptance, engagement, and ownership of interventions.
- National and local health systems and implementing partners should collaborate to complement community engagement and encourage local ownership and effective institutionalization.
- Central government authorities should increase capacity building of district health teams by providing training, delegating power, and providing tools and technologies for implementing iCCM.
- Implementing partners should collaborate with the Ministry of Health and other relevant policy makers to put into place an effective phaseout and eventual exit plan that will ensure program sustainability.

## INTRODUCTION

Compared to the rest of the world, sub-Saharan Africa still suffers from significant under-5 mortality despite making remarkable improvements.^1^^,^^2^ Disparities in under-5 mortality persist among sub-Saharan countries and between the richest and the poorest households.^3^ Infectious diseases continue to account for a sizable proportion of the region’s under-5 mortality. Diarrhea, malaria, and pneumonia combined accounted for approximately 40% of under-5 mortality between 1999 and 2013 compared to the 29% global estimate.^4^ Between 2000 and 2015, pneumonia (16.6%) replaced malaria (16.4%) as the leading cause of mortality among children aged 1–59 months in sub-Saharan Africa.^5^

The United Nations Children’s Fund and the World Health Organization (WHO) recommend integrated community case management (iCCM) of childhood illnesses as a strategy for equitable access to treatment in areas with formal health facility deficiency.^6^ iCCM relies on community health workers (CHWs) using simple algorithms to offer health promotion, disease prevention, and curative services for uncomplicated diarrhea, malaria, and pneumonia.^6^ In 2002, Uganda adopted the Home-Based Management of Fever policy for management of malaria, and, in 2010, adopted the iCCM policy that also introduced CHW training in integrated management of pneumonia and diarrhea within the community.^7^

Appropriate implementation of integrated interventions like iCCM tends to be complex because interrelated yet independent interventions are packaged and delivered together.^8^ The integrated interventions rely on local health system structures and resources for their implementation. Thus, it is important to ensure that the various interventions are packaged in a manner that allow them to be absorbed by the local health system.^9^^,^^10^ Specifically, the interventions need to be institutionalized into the different but interconnected departments through which local health systems deliver health care.^11^^,^^12^ In theory, institutionalization of a health intervention into the health system is believed to occur when the intervention becomes a routinely practiced and integral part of the conventional health system. Thus, institutionalization of iCCM depends on the implementation of a strong iCCM program, which, in turn, requires appropriate CHW training, regular supervision and monitoring, and continuous supply of drugs and commodities among other things.^13^

**Figure:**
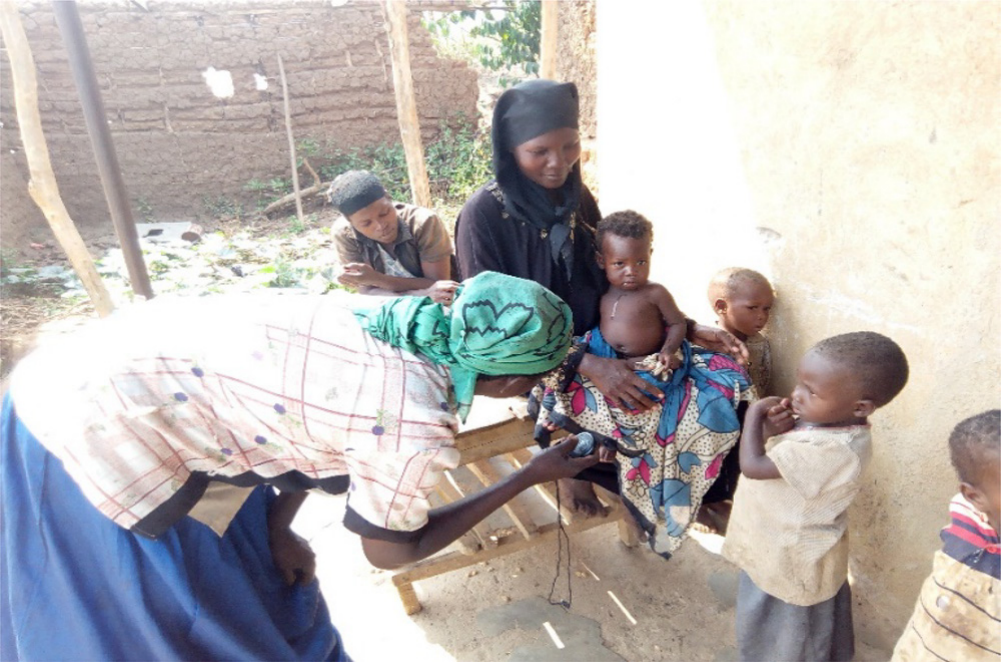
A village health team member counts the respiratory rate of a child who has a cough with fast breathing. Photo credit: © 2013 Edmound Kertho

The local health system in Uganda is based on a decentralized system arising from political decentralization reforms based on the local government system experienced since 1986. Decentralization has been defined as the transfer of some degree of political, administrative, and financial authority from the central government to the local governments.^14^^,^^15^ The local government system consists of administratively nonsubordinated, comprehensive, and judicially accountable local districts. Thus, the decentralized systems were expected to improve access to public services based on community needs, develop local capacity, and enhance transparency and accountability through community participation. In the last 2 decades, districts in Uganda have gradually gained some degree of financial decentralization. To be sure, most local governments are still financed by funds allocated from the central government, although each district has the power to supplement centrally funded budgets with locally generated revenue and approved budgets.^16^

In the health systems context, the decentralization reforms in Uganda meant that the principal roles of the central Ministry of Health (MOH) were redirected to policy formulation, capacity development, planning, inspection, mobilization of resources (such as human resources, health infrastructure, medicines, and other health supplies, as well as health data), and provision of nationally coordinated services including the management of national programs. The national programs run by the MOH include but are not limited to epidemic control, emergency preparedness research, and monitoring and evaluation of health sector performance. On behalf of the MOH, several national autonomous units, including the Uganda Blood Transfusion Service, national medical stores (NMS), various health professional councils, and the National Drug Authority, operate some of the clinical support and regulatory functions.^17^ Consequently, because districts have the overall responsibilities for health service delivery,^18^ to a large extent, the district health team (DHT) operates the local health system. The DHT consists of technical health officials responsible for strategic health planning, management, budgeting, coordination, resource mobilization, and monitoring of overall district health performance.

In the process of institutionalizing integrated health interventions into decentralized health systems, both departments and individuals have often encountered disruptions.^19^^,^^20^ For example, health facility workers with the double burden of providing clinical and administrative services may also be required to take on additional iCCM supervisory roles. Such disruptions may lead to compromised quality of care and delays in achieving equity as local health systems cope with both high disease burden and limited resources.^14^ Previous research has shown that achievement and sustainability of meaningful benefits of iCCM requires adequate health system and political support. Such support is in the form of ongoing CHW training, supportive supervision, provision and replenishment of effective job aids, consistent replenishment of medicines and supplies, and effective approaches for retention and performance of CHWs.^21^^,^^22^ Although the importance of DHT staff empowerment for effective management has been highlighted, the lack of capacity in exercising a decision space for critical health system functions, such as human resource management and quality improvement, has been recognized as a key barrier to health systems performance.^23^ In Uganda, iCCM was gradually introduced into a decentralized health system through the village health team (VHT) strategy that outlines the DHT’s responsibility for overall planning, implementation, and monitoring of VHT activities, and that VHT activities shall be fully integrated into the district health development and operational plans.^24^

**Figure:**
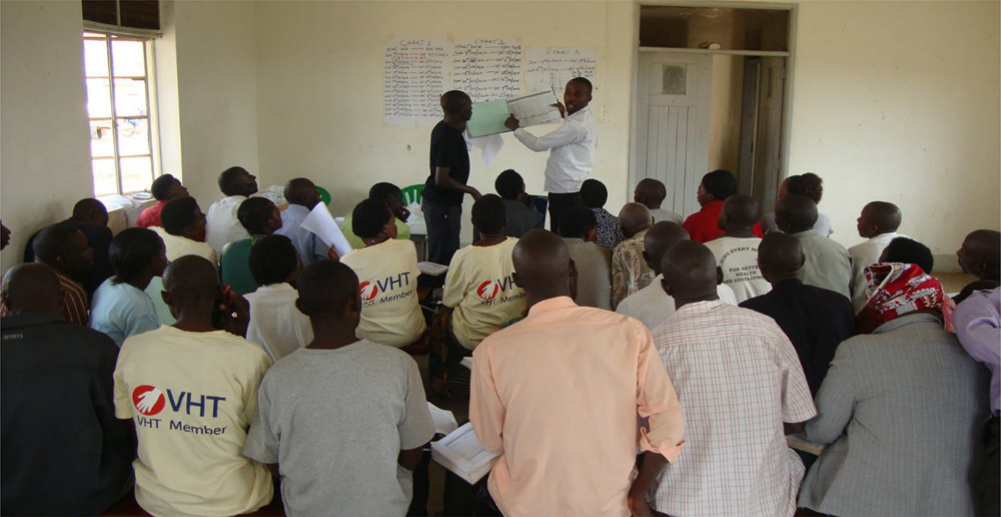
A supervisor shows village health team members how to fill in the iCCM register during a quarterly review meeting. Photo credit: © 2013 Edmound Kertho

We conducted this study to assess the baseline readiness and progress made by DHTs in institutionalizing iCCM into the local health system functions.^25^^,^^26^ Community or organization readiness studies are important in determining whether a program can be effectively implemented and supported by a community.

## METHODS

### Study Design

We used a repeated qualitative assessment consisting of group and key informant interviews in 9 districts in western Uganda. The baseline readiness study was conducted just before the launch of the iCCM policy in 2010 and the follow-up study was conducted in 2015.

### Study Setting

#### iCCM Implementation Context

Since the 2010 launch of the iCCM policy in Uganda, subsequent implementation had been mainly run by implementing partners in collaboration with local district health administrators.^27^ The implementing partners largely consisted of local nongovernmental and international organizations. The national implementation of iCCM was undergoing expansion at the time of the study, and it was estimated to have reached 78 of the 112 districts in Uganda by the end of 2016. There were also government-led efforts to integrate iCCM into national plans and budgets.^27^

### Various Actors’ Roles in Implementing iCCM

#### Malaria Consortium

Malaria Consortium, an international nongovernmental organization, was the main iCCM implementing partner in the study area between 2010 and 2015. It worked closely with the MOH to support and strengthen community-based case management programs for malaria, pneumonia, and diarrhea while emphasizing linkages to formal health services. Malaria Consortium’s approach to the implementation of iCCM followed a health systems strengthening (HSS) approach.^28^ We actively participated in technical working groups and proactively provided progress updates to harmonize the program with national policies without compromising those elements that we believed were essential to achieving a high-quality health care program. These components included supportive supervision, effective referral systems, supply management of medicines and other commodities to minimize stock-outs, as well as strong monitoring and evaluation of the program through routine data collection from VHTs. Thus, we covered several iCCM costs through provision of free diagnostics and medicines, VHT training on iCCM alongside DHT members, job aids, data collection registers, supervision, and behavioral change communication for caretakers and health staff. To support the institutionalization of iCCM, we supported the quantification of iCCM supplies stock at the central level. At the district level, we facilitated learning visits, supported budgeting and forecasting exercises, and supported local supervision of VHTs and data collection through parish coordinators. We undertook all the aforementioned activities under the stewardship of the Maternal and Child Health Division of the MOH and other related divisions. All our activities were geared toward supporting DHTs and the MOH to take local ownership of iCCM and include its costs and activities into their work plans.

#### MOH

The MOH worked closely with key iCCM implementing partners to develop and translate the national iCCM policy and guidelines into practice at the community level. This work was done primarily by developing tools for implementation of different aspects of the policy, such as training materials and curriculum; support supervision forms; registers and data collection forms; and stock management forms. Staff from the MOH acted as trainers and supervisors for iCCM programs. The MOH adopted diagnostic tools and color-coded treatment regimens suitable for the treatment of young children by CHWs in response to advocacy from the implementing partners.

#### DHTs

After the DHT members received training from the Malaria Consortium, DHT members served as iCCM trainers. Together with staff from the Consortium, DHT members conducted training for staff from all levels of health facilities and CHWs. DHT members also acted as supervisors of iCCM to manage the supervision of CHWs by health facility staff and other supervisors from the local government. DHTs collaborated with the Malaria Consortium in digitizing the quarterly reporting of iCCM data into a system known as the District Health Information System II. During the study period, DHTs ensured regular supply of medicines for iCCM through adequate forecasting, quantification, and distribution of medicines. A detailed description of the responsibilities of the health facility workers and CHWs trained follows.

#### District, Local Government, and Health Systems Context

Uganda’s districts are divided into counties, subcounties, parishes, and villages. The village is governed by the local council, which is headed by a chairman. Within the formal health sector structure, a hospital serves a district, a health center level IV serves a county (also known as a health subdistrict), a health center level III serves a subcounty, and a health center level II serves a parish.

A VHT member, also known as a CHW, working from home is considered the health center level I at the village level overseeing between 20–30 households. A VHT comprises 5–6 CHWs who received 5 days of training on disease prevention and health promotion. The community selects 2 VHT members to participate in an additional 6-day iCCM training that is supervised by a health facility worker.^24^^,^^26^ The training prepares the VHT members to classify and treat uncomplicated malaria, pneumonia, and diarrhea using simple algorithms, simple diagnostic tests, and standard treatment regimens that are color coded. It also prepares them to refer sick newborns and children with malnutrition and symptoms of severe illness to higher-level health facilities.^26^ Typically, staff from the nearest health facility supervise and support the VHT members.

**FIGURE 1.**
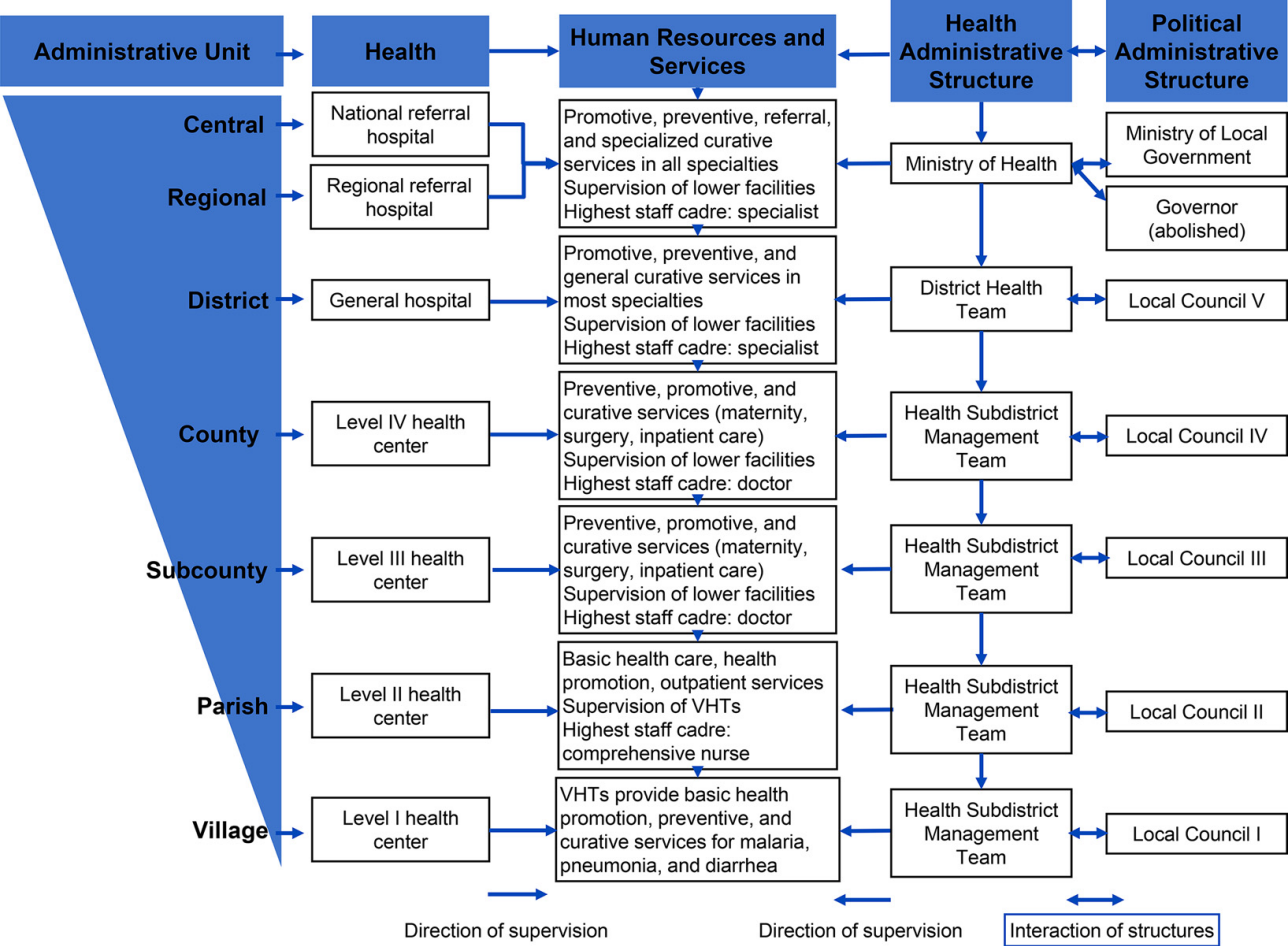
Uganda’s Health and Administrative System Structure

The DHTs forecast the quantities of medicines and supplies that health facilities will need, request the quantities from the NMS, and then distribute the commodities to the lower-level health facilities based on perceived need as assessed by a higher-level facility. The health facility in-charge and the assistant health education officer at the health subdistrict are the custodians of supplies and commodities that the VHTs use, whereas the health facilities at all levels are responsible for replenishment of commodities and health supplies.^24^^,^^26^ Figure 1 illustrates the hierarchy of both health and administrative systems in Uganda.

### Study Sites

Our study drew on data collected from 9 districts in midwestern and central Uganda (Table 1) where the Malaria Consortium (one of the largest implementing partners) had been implementing iCCM since 2009. The districts had an estimated total population of 2.2 million, of which 20% were under-5-year-old children. The districts were served by a total of 276 health facilities, 70% of which were government health facilities, 18% were private not-for-profit, and 12% were private for-profit. The health-seeking behavior of the people in the study is known to vary from treatments at home to both formal and informal health providers outside the home.^29^

**TABLE 1.** Profile of District Health Team and Local Government Members Interviewed on Institutionalizing Integrated Community Case Management, 9 Districts in Uganda

**Readiness Study Group Interview Participants**	**Buliisa**	**Kibaale**	**Kiboga**	**Kyankwanzi**	**Kyenjojo**	**Kyegegwa**	**Kiryandongo**	**Masindi**	**Hoima**
District health officer	X	X	X	X	X	X	X	X	X
District drug inspector	X		X	X	X	X			X
District health inspector	X	X	X	X	X	X			X
District health educator	X	X	X	X	X	X	X	X	X
District surveillance focal person	X	X							X
Health facility in-charge	X	X	X	X	X	X	X	X	X
Health management information focal person	X				X	X			
Malaria focal person	X	X	X	X			X	X	X
Medical superintendent			X	X					
District biostatistician					X	X	X	X	
Key informant follow-up study^a^^b^									
District health officer	N	Y	N	Y	Y	Y	Y	Y	Y
Malaria focal person	Y	Y	N	Y	Y	Y	Y	Y	Y
District biostatistician/HMIS focal person	Y	Y	Y	Y	Y	Y	Y	Y	Y
District planner	N/A	N/A	N/A	N/A	N/A	N/A	N/A	N/A	N/A

Abbreviation: HMIS, health management information system.

a Y means a person was district health team member in 2010 and 2015 and participated in the study.

b N means person was not a district health team member in 2010.

### Study Participants and Data Collection

In 2010, we collected data on readiness of DHTs to institutionalize iCCM into local health systems’ functions during the period that preceded the adoption of the national iCCM policy. Data were collected through structured group interviews held with DHT members in each of the 9 districts (Table 1). In addition, we conducted key informant interviews with selected DHT members who oversaw the implementation of community-based interventions (including iCCM) and the district planners who managed the district budget. The structured interview guides used for both the group and key informant interviews were framed within the WHO health system building blocks. The group interview facilitators asked targeted questions about planned strategies to institutionalize iCCM into local health system functions. Table 2 gives an overview of the health system functions that were explored.

**TABLE 2.** World Health Organization Health System Building Blocks (Functions) Explored in 9 Districts in Uganda

	**Attributes Probed for in the Interview Guide**
Health financing	Sources of funding for health care in general and specifically for iCCM Percentage of the district budget allocated to iCCM Type of providers of child health care services Range of health services provided by both public and private providers and coverage of the health costs at the point of use Most significant change in health financing for children
Service delivery	Scope of child services offered within the district-by whom and when and presence of any performance linked payments The most significant change in health service delivery
Health workforce	Scope of health staffing levels and turnover in the district Scope of VHT coverage and turning over Scope of training, implementation partners, remuneration, motivation. and supervision for CHWs Presence and awareness of iCCM guidelines Most significant in the health workforce
Governance	Extent to which health decisions are made by the DHTs Budget priority setting exercises Health worker and health technology regulation Community participation in health decision making Most significant changes in health governance
Medical products and technologies	Available medicines and supplies for treatment of common childhood illnesses Distribution of the medicines and supplies specifically iCCM medicines Most significant change in distribution of medicines
Information	Management of health data at the local and district level Availability of standardized registers for collecting and submitting community level data Available data submission platforms Community-level indicators fitted into the national HMIS Use of data for decision making at local and district level Key challenges in reporting data at the local and district level Most significant change in the HMIS

Abbreviations: CHW, community health worker; DHT, district health team; HMIS, health management information system, iCCM, integrated community case management; VHT, village health team.

In 2015, the progress made in institutionalizing iCCM into the functions of the local health system was explored by conducting an identical semistructured interview of DHT members and district planners from the same districts. The interviewer asked questions on progress made and the most significant changes observed in implementing various iCCM components into the functions of the local health system.

All group interviews and key informant interviews were conducted in English, audiotaped, and were available as verbatim transcripts. Permission was obtained by a notetaker to both audiotape and write out the discussion that ensued. Permission for the audio recording was also explicitly sought in the consent form that all study participants were required to complete.

In situations when a particular DHT member was not available, the acting or designated person with those responsibilities was interviewed instead. The individuals interviewed in the baseline readiness and follow-up studies were often the same, except for DHT members who had left, been promoted or transferred, or had died. The individuals were purposively sampled based on their roles and the fact that they were believed to have rich information on strategic health planning for the district.

### Data Analysis

Using deductive content analysis, data were explored for patterns related to the different health system functions by the first author (AN). Meaning units were identified directly from what respondents said. These were condensed into descriptive codes that were fitted into the predefined categories relating to the health system components and functions, including financing, service delivery, health workforce, governance, medical products and technologies, and information (Table 2).^30^ The analysis aimed to explore the extent to which iCCM had been institutionalized into the health system building blocks and functions at the district level as proposed by the DHT before the iCCM policy adoption period. The analysis was done using OpenCode 4.0 (University of Umeå, Sweden).

### Ethical Considerations

Ethical approval for the study was obtained as part of the inSCALE study from both the Institutional Review Board of Makerere University School of Public Health (IRB00011353 protocol [100]) and the Uganda National Council of Science and Technology (HS 958). Informed consent was obtained from the study participants.

## RESULTS

In the pre-iCCM policy adoption readiness study, we found that the DHT reported being ready to institutionalize iCCM into their district health systems. They affirmed their readiness by mentioning plans to develop district-specific iCCM activity work plans and budgets; district-led training, motivation and supervision of CHWs; district-led distribution of iCCM drugs and supplies; and advocacy activities for including iCCM indicators into the national health management information system (HMIS). In contrast, in the follow-up study, we found that the level of institutionalization of most iCCM components into the health systems functions ranged from poor to none at all.

Although DHT reported being ready to institutionalize iCCM, follow-up study findings showed the level of institutionalization ranged from poor to none at all.

### Lack of Financing of iCCM

In the readiness study, DHTs suggested that they would ensure sustainable funding for iCCM. They planned to realistically achieve this by avoiding excessive dependency on donor funds. The DHTs often mentioned their plans to advocate for the inclusion of an iCCM budget in district work plans and only mobilize donor funds as supplementary budgets from their district implementing partners. Such supplementary funds would include start-up costs linked to the VHT training among other things.

*We intend to partner with the different nongovernmental organizations working in different localities in our area to train VHTs*. —DHT member, group interview, 2010

The follow-up interviews revealed that financing for iCCM to procure medicines and supplies, train CHWs, and facilitate quarterly supervision meetings remained largely donor dependent. DHTs had difficulties including an iCCM budget in the district work plan and struggled to determine what budgetary lines could be used to cover iCCM-related costs. Most of the district health budget was provided by central government funds to the districts in a lump sum. These funds needed to be distributed among many competing health priorities based on budgetary ceilings. DHTs from across all study districts reported the largest percentage of the central government funds allocated to health were apportioned to health facility running costs. This left meager funds for other activities including community health. The health budget from the central government was supplemented by 13%–16% of locally generated revenue; an insufficient amount to cover the activities in the iCCM district plan. The shortage in locally generated revenue is due to the fact that tax collection in rural areas is often limited and varies every year depending on how much property tax, local service tax service, parking fees, and district rental fees are collected.

*For example [during the budgeting exercise], the health facilities receive the PHC funds [primary health care funds] directly from the central government into their accounts. It is the in-charge of that facility who is the accounting officer together with the subcounty chief. So, they do their work plan and budget. We only do the supervision to see whether they comply with what is in their work plan. Health facilities also have health management committees. They [health facility leadership] look at previous budget performance as they bring the management committee on board, and we are encouraging them to bring VHTs on board too. From there, the budgets go to the health subdistrict from where they are approved.* —District health inspector, interview, 2015

Although no concrete iCCM financing plans existed in most of the districts, DHT members interviewed in the postadoption period conceded that a well-running iCCM program was beneficial and could lead to decongestion of health facilities and improved health-seeking behavior in the communities.

*Generally, [iCCM] improved service delivery and queues shortened at the health facilities. The few health workers were not burdened by the workload anymore at health facilities … and the blood test [malaria rapid diagnostic test] is now more available and demanded for … so I think those are some of the changes.* —District health officer, interview, 2015

### Lack of Institutionalization of iCCM Medicines

Overall, the post-iCCM policy adoption findings were not in line with procurement and supply management system plans indicated by the DHTs in the readiness study. DHTs had previously proposed advocating for the supply of iCCM drugs and supplies through the NMS with a push system for medicines in hard-to-reach areas. The DHTs reported that their plans for procurement were based on some of the lessons that they had learned during the implementation of the preceding Home-Based Management of Fever national program. DHTs recognized that the availability of medicines and supplies acted as a motivating factor for VHTs, but drug stocks-outs did the opposite. They often mentioned that demotivation among VHTs due to drug stock-outs often manifested as concurrent neglect of the regular health promotion and education activities among some VHTs.

*… with regard to motivation, when Coartem is present, the VHTs are very active. We shall do whatever is within our power to ensure full-time stocking of drugs and supplies, but then again, this factor [availability of drugs] is greatly dependent on dynamics within the NMS [in reference to inefficiencies in the wider procurement and supply cycle].* —DHT member, group interview, 2010

Despite this assertion, half a decade post-iCCM policy adoption, the delivery of medicines and commodities to the community level came to a complete halt in 8 of the 9 districts after departure of the implementing partners.

*We do not have a budget line for [iCCM] as a district. It is also quite complex to requisition for drugs. NMS has a policy not to distribute medicines to the community, and the VHTs are considered not professionally trained to handle medicine. NMS considers only health center II and health center III. There are also no clear guidelines on how to requisition VHT commodities from NMS.* —Malaria focal person, interview, 2015

Overall, the DHTs felt that they lacked stewardship, clear instructions, and direction from both the implementing partners and the MOH on how best to transition from externally supported implementation to district-led programming. More elaborately, the DHTs had no clear guidelines or directives from the MOH on how to transition to a district-led community medicine distribution policy. There were also conflicting community medicine distribution policies that were prone to different interpretations by the various DHTs. These included the iCCM guidelines, which allowed for the prescription of amoxicillin at the community level, and the National Drug Redistribution Policy, which allowed for the redistribution of medicines and commodities between different levels of health facilities in cases of shortages but not the prescription of antibiotics at the community level. Due to these conflicting guidelines, and the complete absence of standard accountability tools at the community level, the integration of iCCM medicines and commodities into district procurement chains was not perceived as a viable option for most DHTs.

DHTs felt they lacked stewardship and clear instructions on medicine distribution.

### Successful Institutionalization of iCCM Medicine Procurement

Despite the conflicting community medicine distribution guidelines, the follow-up study showed that 1 district used the National Drug Redistribution Policy to provide iCCM services in at least 2 of its 4 subcounties for the start with intent to expand to all its subcounties. The 2 subcounty implementation phase was a DHT-led demonstration project (pilot) undertaken to understand the best approaches for transitioning to an independent district-led iCCM program. However, the demonstration project also faced several challenges as the DHT had to develop a robust accountability system for distributing community-level medicines and commodities on its own. The system consisted of collaborative efforts between the head of the political domain at the village (local council chairman) and VHTs. Through this system, VHTs were able to requisition for and receive drugs from the health facility under the witness of the local council level I chairman who was required to append a signature on both the requisition and delivery (receipt) forms. Thus, medicines and commodities redistributed to the community levels were accounted for as stock used by the outpatient department. The arrangement was well received by the community and had managed to avert interruptions in program implementation by the time of the study.

*Actually, NMS has not supplied [iCCM drugs and supplies]. They promised, but they have not started. [NMS] were saying that they have not quantified what is needed and are still sorting out some policy issues … but initially, with our partners’ support, the VHTs used to get the drugs during quarterly meetings or at times the health workers would take the drugs to them. Now, in case of a drug shortage, there is a form VHTs fill out and write a requisition form for drugs in the presence of the chairman local council level I who also signs the form that is taken to the health facility in order to receive drugs. It is a local arrangement that we are trying out.* —Malaria focal person, interview, 2015

One more district mentioned its intent to pilot district-led iCCM service delivery in 1 subcounty in the near future.

*We want to interest our implementing partners to come back because we have gaps. Presently, there are no drugs in the communities [for the VHTs] because the government has not incorporated iCCM into its policies. We informed the MOH during workshops or whenever there was a chance. The MOH okays us to go ahead and implement iCCM, but they do it verbally, so we fear to implement it unless it is put in writing. However, there are some health centers where we are piloting [commodity distribution from the health facilities’ stocks].* —District health educator, interview, 2015

### Increased Availability and Strengthened Human Resources for iCCM

DHTs reported that the percentage of VHTs trained on the basic package per subcounty compared to that required by the national VHT strategy increased from a range of 0%–64% pre-iCCM to more than 90% in all study districts in the follow-up study. This increase was attributed to the concerted efforts of nongovernmental district implementation partners. In the readiness study, DHTs suggested that they would include VHT training budgets in their work plans, orient health facility staff on iCCM, and develop concrete supervision and motivation plans in accordance with MOH protocols, as some of the key strategies for retaining VHTs. They believed that the training led by nongovernmental organizations was more aligned to individual nongovernmental organization goals than district interests and should thus gradually be weaned off.

Percentage of VHTs trained on basic package increased to more than 90% in all study districts.

*We have different VHTs with different motivating factors and different training. For example, X [name of organization withheld] trains VHTs on HIV, and XX [name of organization withheld] trains them on onchocerciasis … VHTs trained by XXX are motivated because they receive motivators like umbrellas and gumboots; partners need to cooperate to harmonize VHT activities and remuneration as people working toward a common goal which is improving community health in line with MOH guidelines.* —DHT member, group interview, 2010

Contrary to the readiness study, the follow-up study showed that there were no clearly defined district-led training, motivation, and supervision plans for the VHTs. DHTs were not yet prepared to transition from partner-led to district-led activities. The motivation and supervision of VHTs was happening on an ad hoc basis as it had initially depended on implementing partners for financial and technical support. The DHTs reported that once the implementation partners left, the supervision became increasingly irregular and infrequent. Remarkably, in the majority of the study districts, supervisors encouraged VHTs to form self-help groups and engage in income- generating activities such as savings and credit cooperative organizations that could improve motivation without necessarily depending on district resources.

*It [district-led motivation of VHTs] has been somehow minimal. Of course, our VHTs have been having a high interest to perform but have been experiencing demotivating factors like stock-out of drugs. However, the district receives drugs from NMS. So as a district, I cannot say we have a plan to retain these VHTs; it also depends on their functionality. Actually, what we are doing is that we integrate them in our health system, for example, by asking them to do services like mass mobilization.* —Malaria focal person, interview, 2015

### Lack of Institutionalization of Community Data into the HMIS

In the readiness study, DHTs often acknowledged a need for improved reporting and use of community-level data for district planning on a routine basis. The DHTs proposed advocating for harmonization of community-level data collection tools with the HMIS (since some community- level indicators were not being captured by the existing HMIS forms) and emphasized the importance of VHT training on registers, timely provision of registers, and coordination and facilitation of data submission as some of the strategies for improved community-level data submission.

*… they [VHTs] received training in theory and were provided with the registers later on. They did not know how to fill the registers properly, and this frustrated not only them but also the records staff.* —DHT member, group interview, 2010

However, in the follow-up study, community-level data reporting did not align with the improvements that had initially been suggested by the DHTs in the readiness study. Community-level data were submitted from health facilities to the district level every quarter because monthly submissions were considered cumbersome and unpractical. The quarterly report submitted captured absolute numbers of children treated for specific diseases but not the medicines dispensed to the children. According to some, this was a consequence of some implementing partners providing VHT registers and reporting forms that did not match the indicators captured by the HMIS.

*At first, the data were managed by the donor, but later it was entered in HMIS after we got pressure from the government. We have just completed entering the iCCM data from 2009 up to last year. The donor’s tool was not matching with the HMIS data base. Now, the HMIS 097 is the quarterly reporting form for iCCM.* —District biostatistician, interview, 2015

### Lack of Coordination and Governance of iCCM

Although nearly half a decade had elapsed after the national adoption of the iCCM policy, the DHTs did not clearly mention any effective strategies had been used for policy implementation on the ground. iCCM had not been comprehensively institutionalized into district-specific work plans with several requirements that would have otherwise led to its effective implementation reported to be lacking. The existing national policies did not offer guidance on how to transition from partner-led programs to district-led ones, and the guidelines that existed did not allow DHTs enough formal decision-making autonomy regarding budgeting and implementation. This was manifested at several functional levels of the health system: (1) finance and expenditure allocations were based on government-set ceilings that limited the allocation of funding to iCCM activities, (2) DHTs were uncomfortable to make decisions concerning the drug supply for community medicines that resulted in drug stock-outs, and (3) DHTs were not able to make decisions about health information system reporting to the central level. Nonetheless, there was a high level of awareness of iCCM in the districts, and there were plans to include VHTs in the district budget priority-setting exercises in some of the districts.

These findings were not concordant with the level of commitment to develop comprehensive district-specific work plans for iCCM that was expressed in the readiness study when DHTs had proposed that comprehensive work plans would encompass consolidated community sensitization plans, VHT supervisions plans, and VHT motivation plans to sustain implementation.

*We have no VHTs at the moment. We have a DHT. We plan on recruiting enough staff at the health facility level. We have made iCCM known to the health subdistricts, and we shall make it known to the community. We are anxious for the program and our low VHT-coverage does not stop us from embracing the program.* —DHT member, group interview, 2010

## DISCUSSION

Implementing a well-functioning iCCM program is an HSS intervention that touches on all areas of the health systems framework including workforce development, establishment of community information systems, service delivery, and improvements in the procurement of medical supplies and technologies.^28^ Although we took an HSS approach as implementing partners, our findings show that iCCM implementation remained largely noninstitutionalized into district-specific work plans. Specifically, there was neither evidence indicative of the integration of iCCM color-coded medicines into health facility procurement supply chains nor the integration of budget lines for training, supervision, and motivation of VHTs into district work plans. Only 1 district made significant strides in achieving district-led iCCM implementation by ensuring a functioning procurement and supply chain for iCCM medicines and supplies.

Perceived barriers to institutionalization of iCCM at both the district and national levels included lack of proper stewardship on how to transition from nongovernmental organization to district-led implementation, lack of agreement of national guidelines on community-level drug distribution and poorly established community-level accountability systems, lack of integration of some of the iCCM indicators in the national HMIS, lack of integration of iCCM medicines into the national drug supply chain, limited funding from the central government, and the lack of agency (capacity to act independently and make their own free choices) among DHT members.

The challenges experienced by the DHTs in Uganda resonate with those documented in iCCM programs elsewhere in sub-Saharan Africa.^31^^–^^33^ These findings underscore the missing link between strengthening of a community health systems in relation to the wider health system. Strengthening of the community-based system in isolation from the higher levels of the health systems is prone to fragmentation and inefficiency. For example, it is more beneficial to strengthen the entire health system supply chain than to focus on the community supply chain alone. Similarly, the lack of harmonization between iCCM monitoring indicators with those captured by the national HMIS can lead to challenges in monitoring important outcomes, such as disease trends and the contribution of CHWs in managing key illness. Such outcomes are crucial for identification of program needs, advocacy, budget allocation, and fundraising.^34^ Furthermore, studies commissioned by the iCCM task force in Africa and elsewhere emphasize the need to develop a minimum set of essential indicators for iCCM at the national level.^35^ However, generating these indicators would require closer collaboration between MOH units, particularly those responsible for malaria and other infectious diseases and child health. Limited central-level financing is likely to affect subsequent training of VHTs and support supervision. The lack of community-level accountability tools such as documents that clearly capture how medicines are transferred from health facilities and are subsequently distributed to specific VHTs is inherently connected to drug stocks at the community level. This finding has significant bearing given the overwhelming body of evidence on the negative effects of drug stock-outs on the motivation of VHTs in Uganda.^36^^–^^38^ Thus, current recommendations arising from WHO consultation meetings and the general literature on iCCM discourage implementation of community programs in silos by requiring that primary health care strengthening programs target both community and the higher levels of care.^39^^–^^41^

Lack of community-level accountability tools to capture how medicines are distributed to VHTs contributes to drug stock levels.

Within Uganda’s decentralized health system, DHTs are more or less autonomous segments of the national health system, consisting of all recognized health sector actors whose activities should be reflected in the district health plan.^18^ The MOH has the role of a principal agent with the mandate to encourage local institutions, such as the DHTs, to make choices that achieve the objectives of the national health system. Such obligations include the monitoring of local officials and the use of incentives and rules to shape local decisions.^16^ Likewise, at the local level, DHTs are supposed to have a decision space that allows them to make a range of choices regarding the operation of health services within the district health system. Among other things, the custodial roles of DHTs include planning for and management of health services delivery in addition to implementation of policies. However, the effectiveness of the decision-making powers given to DHTs with respect to iCCM also depends on individual and institutional capacities provided by the MOH through provision of guidelines and training. This is important as a couple of studies have demonstrated that synergy between decision space, capacity, and accountability promotes good decision making at lower levels of decentralized systems.^42^ Given their perceived decision-making power, local authorities may make innovative choices that are different from the directed change that the MOH imposes on them through its central authority.^16^ However, our findings are in concordance with other studies, suggesting that poorly coordinated policies within decentralized health systems tend to render themselves to different interpretations based on perceived agency.^43^ The findings are also indicative of a general perceived lack of empowerment (in terms of clarity of the boundaries of power entrusted to them and available implementation tools) among DHTs with respect to making innovative locally beneficial decisions around the institutionalization of iCCM. This was regardless of the fact that we had used an HSS approach to implementation. The perceived lack of agency among DHTs as opposed to the initial perceived readiness points to factors within the wider health system that are sometimes beyond the DHTs’ control such as conflicting national guidelines and limited central funding.^44^^,^^45^ The reported decision-making capacity gap has also been observed in other studies.^23^^,^^46^ For example, a study by Alonso-Garbayo et al (2017) described the varying levels of agency exhibited by district authorities as (1) often operating within close boundaries defined by public policy, (2) occasionally making decisions beyond their conferred authority in some management domains, and (3) not being able to use all decision-making powers allocated to them in other domains.^46^

We identified some of the enabling factors for institutionalizing iCCM as illustrated by the district that showed evident progress, included a strong sense of autonomy, leadership, and coordination. Cooperation between the DHT and other local government administrative structures led to the establishment of a local accountability procurement system for iCCM medicines. The innovative collaborative efforts increased community acceptance and ownership of the program. The example is a typical demonstration of the effects of context factors outside the health system (such as political leadership) on implementation of health interventions.^12^

Moving forward, we concur with the wider institutionalizing community health body of practice recommendations stipulating that country expansion of iCCM programs in the hard-to-reach areas where they are most needed, among other things, requires tackling persistent challenges to institutionalization of iCCM that have been identified over the years. Such challenges include but are not limited to ineffective designing of national and local policies that are not based on the best available evidence, ineffective drug supply chains, and insufficient supervision and motivation of CHWs.^47^ It is also a generally recommended good practice that local communities (such as DHTs and the people they serve) should be engaged in the designing of health interventions and implementation approaches at an early stage. Community engagement should be complemented with strengthened collaboration between national and local health systems and implementing partners to enhance local ownership and effective institutionalization of iCCM. This kind of approach will foster the development of country- and district-specific work plans addressing priority issues while harmonizing donor support around the issues.^47^

Finally, empowerment of DHTs requires capacity building in terms of provision of necessary training, delegation of power, and provision of tools and technologies for implementation of iCCM from central government authorities. Lack of necessary implementation tools and accountability mechanisms is inherently known to affect the degree of exercising decision space.^43^^,^^48^ This will subsequently enhance their performance and thus effectiveness in the institutionalization of government initiatives at the local district level. From the implementing partner perspective, it is essential to have clear plans to ensure smooth transitioning from partner-supported programs to fully institutionalized and locally run programs right from the onset of program implementation. Subsequently, necessary steps must be taken to ensure a phased exit while strengthening DHTs and national government to take complete ownership for the financing and management of iCCM programs. Progressively, Uganda has developed a community health roadmap and several guidelines have been harmonized since our study was conducted. We recommend further research to assess program maturity under these new circumstances.

### Limitations

This study has some limitations. Given that the sample districts are all from mid- and central-western Uganda, the study findings may not necessarily be generalizable elsewhere. Our study only assessed readiness from the perspective of the DHTs using the health systems framework without making objective system-wide readiness assessments. However, to the best of our knowledge, there were no specific iCCM program effectiveness assessment tools at the time of our study. Several tools that can be used to assess the readiness of community health programs such as the CHW assessment improvement matrix have since been developed.^49^ Still, the tools that have been developed are not specific for iCCM. Nevertheless, subsequent studies should use such tools to identify specific areas of weakness where additional focus and resources may be needed before implementation. There was limited turnover of some DHT members. However, this problem is not expected to affect study results greatly as there was no instance where an entire DHT was entirely replaced. Also, the study did not explicitly explore the varying levels of pre-iCCM functioning VHT members, number and nature of community health implementing partners in each district, and factors outside of the health sector. Yet, these factors could have had some effects on the extent of iCCM institutionalization.

## CONCLUSION

Despite limited health resources and health systems challenges, under the leadership of Uganda DHTs, several small steps have been taken to institutionalize iCCM into the routine function of the district health systems. Sustainable scale-up of the known potential benefits of iCCM requires high-level cooperation and policy alignment among key actors at the national level including the MOH, the NMS, and the National Drug Authority. Prior engagement with DHTs is necessary to ensure their buy-in before introducing the intervention to ensure district-led and locally owned institutionalization. Implementing partners should only play a supportive role with a clearly outlined handover and exit plans. Collaboration between various administrative, political, and community sectors is necessary to build effective well-institutionalized systems. Central to prompt and effective integration of national iCCM policies into district health management systems is the need for the MOH to provide sufficient leadership in terms of strategic policy direction formulation, ensure capacity building for DHTs through training and provision of adequate tools for implementation, and establish sustainable partnerships with government and nongovernmental organizations working with iCCM.

## Notes

**Peer Reviewed**
